# Improving energy efficiency of electrochemical blackwater disinfection through sequential reduction of suspended solids and chemical oxygen demand

**DOI:** 10.12688/gatesopenres.12873.2

**Published:** 2019-01-23

**Authors:** Brian T. Hawkins, Tate W. Rogers, Christopher J. Davey, Mikayla H. Stoner, Ewan J. McAdam, Brian R. Stoner

**Affiliations:** 1 Center for WaSH-AID, Duke University, Durham, NC, 27701, USA; 2 Department of Electrical and Computer Engineering, Duke University, Durham, NC, 27701, USA; 3 Triangle Environmental Health Initiative, Durham, NC, 27701, USA; 4 Cranfield Water Sciences Institute, Cranfield University, Cranfield, Bedfordshire, MK43 0AL, UK

**Keywords:** Sustainability, water reuse, electrochemical disinfection, ultrafiltration, activated carbon, blackwater

## Abstract

Onsite reuse of blackwater requires removal of considerable amounts of suspended solids and organic material in addition to inactivation of pathogens. Previously, we showed that electrochemical treatment could be used for effective pathogen inactivation in blackwater, but was inadequate to remove solids and organics to emerging industry standards. Further, we found that as solids and organics accumulate with repeated recycling, electrochemical treatment becomes less energetically sustainable. Here, we describe a pilot study in which concentrated blackwater is pretreated with ultrafiltration and granular activated carbon prior to electrochemical disinfection, and show that this combination of treatments removes 75-99% of chemical oxygen demand, 92-100% of total suspended solids, and improves the energy efficiency of electrochemical blackwater treatment by an order of magnitude.

## Introduction

Electrochemical disinfection is a promising approach to sustainable decentralized waste water treatment because it enables oxidative inactivation of pathogens without requiring onsite storage of disinfecting chemicals (e.g., sodium hypochlorite or chlorine gas). In systems that utilize recycled blackwater for flushing, these processes become more energy intensive over time as solids accumulate in the process liquid ^1^. Understanding how the constituents of blackwater that accumulate in such systems contribute to the decreased efficiency of electrochemical disinfection is key to developing remediation strategies that will enable practical implementation and long service lifetimes.

Previously, we investigated the effects on electrochemical disinfection energy efficiency of removing total suspended solids (TSS) with improved settling tank design ^2^ and removing chemical oxygen demand (COD) with granular activated carbon (GAC) ^3^ and found that only the latter resulted in a significant improvement. This implied that soluble COD was the principle cause of diminishing efficiency with repeated recycling of blackwater. However, because we had not completely removed TSS in any of these studies, we could not conclude definitively that suspended solids did not contribute. We also found that the same GAC media could remove a substantial fraction of blackwater COD in multiple treatment batches—suggesting that the filter medium was not fully saturated in these experiments—but that within each batch up to half of COD could not be readily removed by GAC ^3^. Thus, we hypothesized that this poorly adsorbing fraction of COD was associated with suspended particulate matter not removed by settling or GAC, and further, that successful removal of this fraction from blackwater would improve the energy efficiency of subsequent electrochemical disinfection. We tested this hypothesis in a pilot study in which blackwater was treated by cross-flow ultrafiltration followed by a GAC packed bed filter, and assessed the effect of these combined pretreatments on the energy required for subsequent electrochemical disinfection.

## Methods

Blackwater was collected from a prototype blackwater recycling toilet system previously described ^2^. Procedures for the collection of human urine and feces used to generate blackwater were reviewed and approved by Duke University’s Institutional Review Board. Characteristics of the untreated blackwater used in this study are shown in Table 1.

**Table 1.** Characteristics of untreated blackwater.

Parameter	Range
Total solids (mg / L)	2001 – 2634
Total suspended solids (mg / L)	180 – 667
Turbidity (NTU)	248 – 461
Color (Pt/Co units)	1600 – 1800
pH	8.88 – 9.02
Most probable number (# / ml)	1.1 × 10 ^8^ (all)
Chemical oxygen demand (mg / L)	864 – 1818

NTU: Nephlometric Turbidity Units

Ultrafiltration was carried out in 8–12 L batches by passing blackwater through an ultra-high molecular weight polyethylene tubular membrane with a nominal pore size of 0.02 µm and a total active surface area of 0.07 m ^2^ (Porex, Norcross, GA, USA) with a centrifugal pump (Lowara, Montecchio Maggiore, Italy) run in a recirculation configuration. In these experiments, flow was maintained between 28 and 30 L min ^-1^ for a cross flow velocity in the retentate channel of 3.7 – 3.9 m s ^-1^. Transmembrane pressures were monitored by pressure transducers (Omega PX039-015G5V, Omega, Norwalk, CT) on either side of the membrane connected to an Omega OM-DAQ-USB-2400 data logger, and during ultrafiltration typically ranged between 2 and 2.5 bar. Transmembrane flux was monitored by placing the permeate collecting vessel on a balance connected to a computer and using ADAM DU software to log changes in mass, and during ultrafiltration typically ranged between 80 and 120 kg m ^-2^ h ^-1^ ( Figure S1).

Ultrafiltered blackwater was passed through a packed bed column filter with Aquacarb® 830, an 8 × 30 mesh-sized GAC derived from bituminous coal (Evoqua, Pittsburgh, PA), as the medium. The filter consisted of 1.8 kg GAC in a PVC pipe (9.4 cm inner diameter) with a media length of 58 cm, a media volume of ~ 4 L, and an interstitial volume of ~1 L. Liquid was pumped through at a rate of ~1 L min ^-1^ in a recirculation configuration for up to 18 h. In a subset of experiments, COD was monitored during the first ~2 h of GAC treatment to evaluate the COD removal kinetics of GAC with ultrafiltered blackwater in comparison to untreated blackwater ( Figure S2).

Electrochemical disinfection was performed as previously described ^3^ in an 8-L HDPE tank with a commercially available electrochemical cell (Hayward Salt&Swim 3C) run at 24 VDC. This process effects disinfection by oxidizing chloride (primarily from urine) into chlorine. Measurements of water quality parameters were performed as previously described in detail ^1– 4^. Bacterial inactivation was assessed with a 3-well most probable number (MPN) method using lysogeny broth (LB) for dilution and culture as previously described in detail ^1– 4^. Disinfection was defined as reduction of MPN to < 5 / ml; energy required to achieve this level of disinfection was calculated as previously described ^1^ and shown in Figure S3.

Statistical analyses and visualizations were performed using GraphPad Prism v7.04.

## Results and discussion

**Figure 1.**
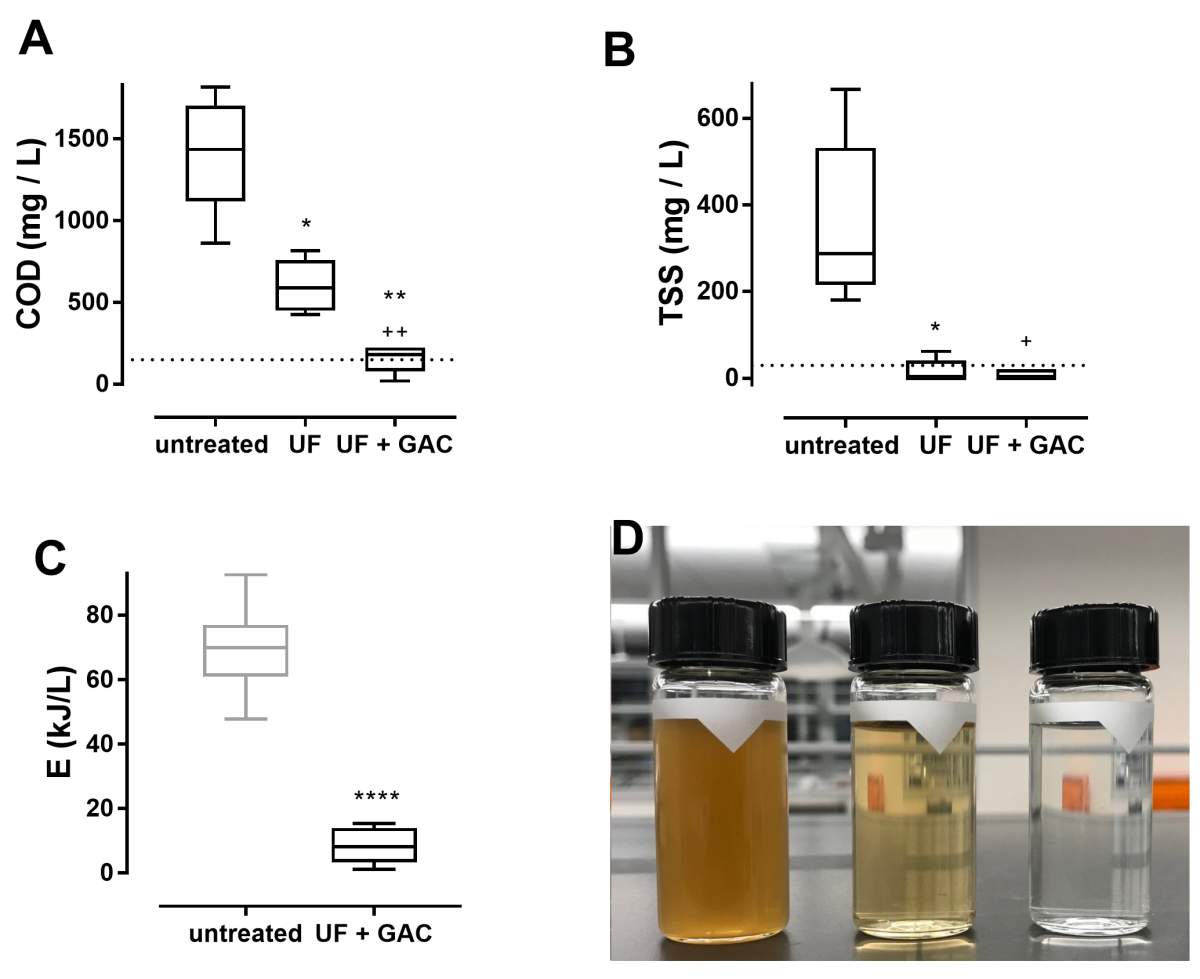
Pilot study combining ultrafiltration (UF) and granular activated carbon (GAC). **A** and **B**: improvements in chemical oxygen demand (COD) and total suspended solids (TSS), respectively, with UF followed by GAC. Data are n = 5 batches, measurements taken sequentially in each batch. Dotted lines indicate ISO 30500 category B standards (150 mg/L COD and 30 mg/L TSS). Significance determined by repeated measures one-way ANOVA with a Tukey’s multiple comparisons test. * = adjusted p < 0.05 vs. untreated, ** = adjusted p < 0.01 vs. untreated, ^+^ = adjusted p < 0.05 vs. UF, ^++ ^= adjusted p < 0.01 vs. UF. **C**: Comparison of energy required to achieve disinfection (MPN < 5/ml) of blackwater treated by UF and GAC prior to electrochemical disinfection (n=5) to untreated blackwater (n=18). Untreated data are from a previous study ^3^. Significance determined by two-tailed t-test, **** = p < 0.0001. **A**– **C**: lines indicate median, boxes 25 ^th^ and 75 ^th^ percentiles, error bars maximum and minimum values. **D**: Samples of untreated, UF treated, and UF + GAC treated blackwater (left to right).

Results are presented in Figure 1. Ultrafiltration significantly reduced blackwater COD by an average of 55% (range 32–74%) and TSS by an average of 97% (range 92–100%). Subsequent treatment with GAC was associated with further reduction of COD to near or below the ISO 30500 category B standard (150 mg/L) for an average total COD reduction of 87% (range 75–99%). These reductions in COD and TSS were associated with a reduced energy demand for the electrochemical process to achieve the desired disinfection threshold to an average of 8.5 kJ/L, which represents an order of magnitude improvement compared with the same process using untreated blackwater (70 kJ/L).

For the purposes of this study we defined disinfection as MPN < 5 / ml, as we have found little to no bacterial regrowth in blackwater treated beyond this threshold. Since many species of bacteria grow in suspension in LB, including coliforms, this method and threshold serve as a conservative estimate of treatment required to inactivate pathogenic bacteria in blackwater. However, an important limitation of this study is that we did not assess the ability of this process for inactivation of viruses, protozoa, or helminth eggs. Future studies on this process will address specific pathogen removal, in particular the surrogates indicated by the recently published ISO 30500 standard ^5^.

Treating blackwater first with ultrafiltration, then with activated carbon, followed by electrochemical treatment, has specific advantages. The removal of suspended solids by ultrafiltration appears to allow for faster adsorption of soluble species by subsequent GAC treatment, which could make GAC treatment in a single-pass configuration practical and thus eliminate the need for a recirculating pump (Supplemental Data, Figure S2). Further, the removal of suspended solids minimizes the tendency of the GAC packed bed filter to clog, thus obviating the need for frequent backwashing.

Cross-flow ultrafiltration requires considerable energy input to the pump to achieve the necessary cross-flow velocity needed for practical membrane productivity. The test rig used for these studies uses a ¾ horsepower-rated pump, and runs at ~850 W when processing blackwater. Based on the runtimes of each trial ( Figure S1) we estimate the energy cost for ultrafiltration in these studies to be 391 ± 60 kJ/L, which is significantly more than the 62.5 kJ/L gain in energy efficiency realized in the electrochemical process ( Figure 1C). It is important to point out, however, that we have not yet optimized this process for the treatment of blackwater, and that an in-depth study of optimal running parameters (cross flow velocity, transmembrane pressure, and membrane surface area) is expected to yield a more efficient process.

Furthermore, the reductions in TSS and COD with the combination of ultrafiltration and GAC far exceed anything we have achieved with electrochemical oxidation alone. We’ve run the electrochemical process used here on untreated blackwater for considerably longer than is required to achieve disinfection, and found that energy expenditures greater than what the unoptimized ultrafiltration process requires (471 – 575 kJ/L) only resulted in 32–38% reduction in COD and no significant reduction in TSS. Thus, while ultrafiltration adds complexity to the system and increases the overall energy demand compared with electrochemical oxidation alone, this is likely an unavoidable tradeoff in order to treat blackwater to ISO 30500 effluent standards.

This pretreatment regime allows for shorter runtimes on the electrochemical process, which will prolong the service lifetime of the electrodes. Similarly, disinfection of pretreated blackwater is achieved with much lower free chlorine concentration-time product (CT) (< 50 mg min/L) compared to untreated blackwater (which can require CT in excess of 2000 mg min/L to be disinfected) ^1^. This reduces the duration of time system components (plumbing, tanks) will need to be in contact with the highly oxidative chemistry of the process liquid and thus increase their service lifetime. 

## Conclusions

Further optimization of the component processes to minimize energy and capital costs and a more thorough assessment of component life cycle and efficacy in specific pathogen removal are necessary to reduce this approach to practice. Although these results are preliminary, we believe they serve as a proof of concept for a practical approach to onsite blackwater treatment that will meet emerging industry standards.

## Data availability

Raw datasets are available on OSF, project “Improving energy efficiency of electrochemical blackwater disinfection through sequential reduction of suspended solids and chemical oxygen demand”, https://doi.org/10.17605/OSF.IO/GRMJT ^6^.

Data are available under the terms of the Creative Commons Zero “No rights reserved” data waiver (CC0 1.0 Public domain dedication).

## Supplementary material

**Figure S1: Ultrafiltration runs.** Shown are plots of the transmembrane flux (in red) and transmembrane pressure (TMP, in blue) for each of the five ultrafiltration runs in this report.

.

**Figure S2: Effect of ultrafiltration of COD removal by GAC.** Shown are plots of COD in one experiment with untreated blackwater and two with ultrafiltered blackwater. Blackwater was treated in 8-L batches by continuous recirculation at ~1 L min ^-1^.

.

**Figure S3: Disinfection curves and energy calculations.** Shown are plots of each electrochemical disinfection run following ultrafiltration and GAC treatment. Black dotted line indicates the disinfection threshold (MPN < 5/ml), red dotted line indicates the disinfection energy (E _d_) interpolated from where the plot crosses the disinfection threshold.
