# Supplementary figures and images for: Improving energy efficiency of electrochemical blackwater disinfection through sequential reduction of suspended solids and chemical oxygen demand
Source: Gates Open Res. 2019 Jan 23;2:50. Originally published 2018 Oct 5. [Version 2] doi: 10.12688/gatesopenres.12873.2 (PMC6350407; doi:10.12688/gatesopenres.12873.2)

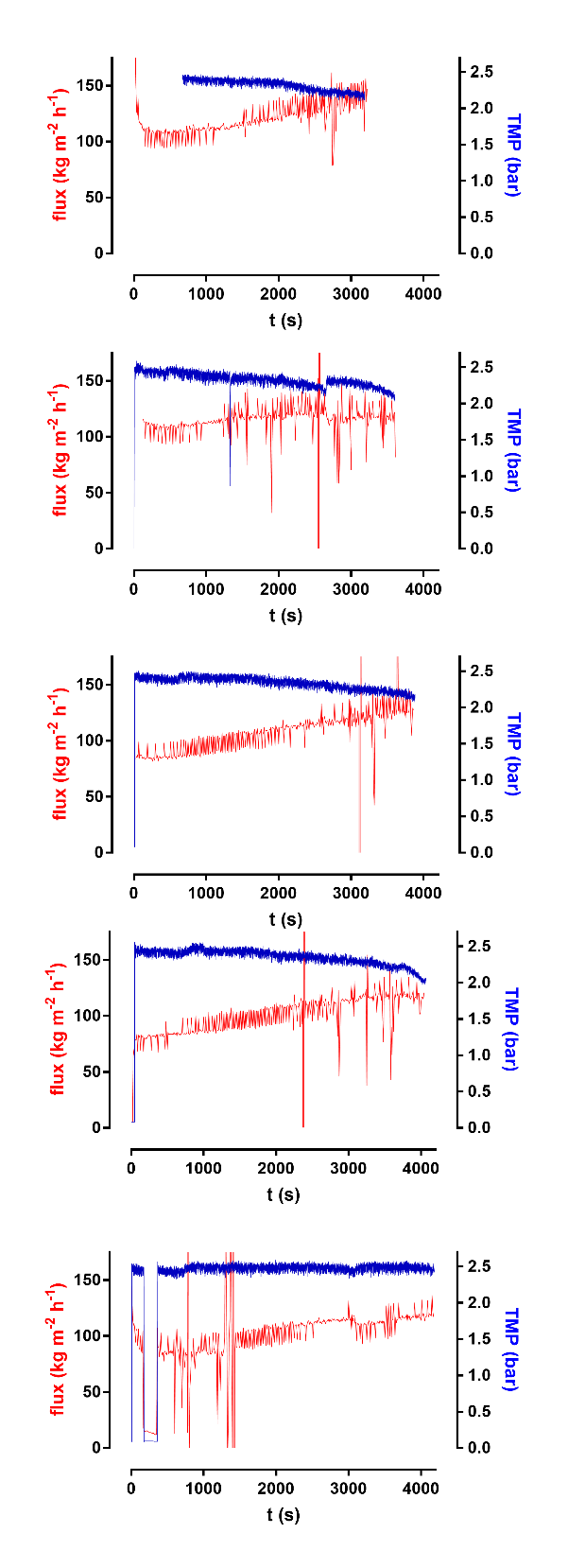

Supplement: Supplementary file 1 [file gatesopenres-2-14010-s0000.tgz › f6a3f83f-06b3-4a80-8f3d-00bd08546858_Figure_S1.tif]

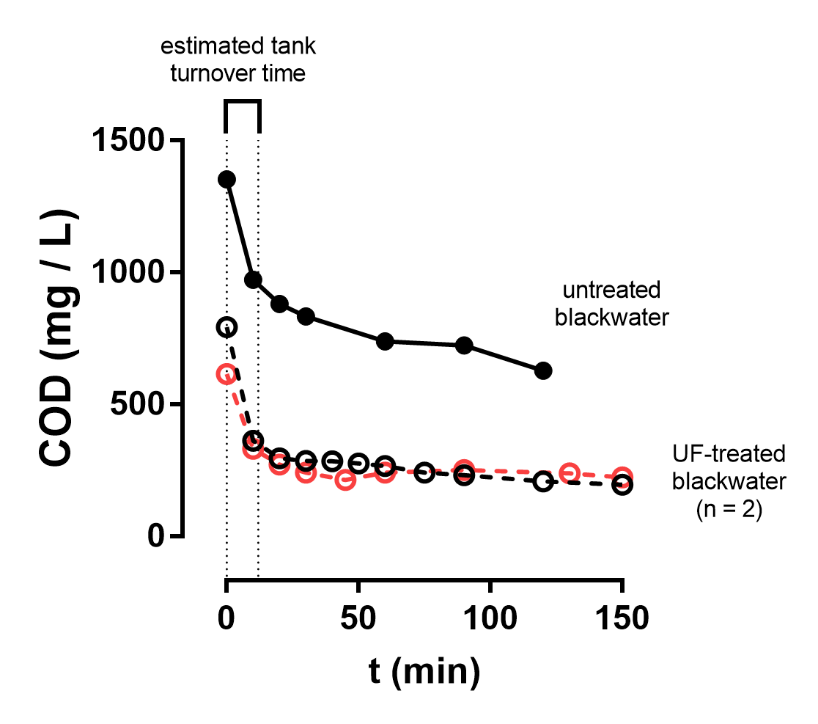

Supplement: Supplementary file 2 [file gatesopenres-2-14010-s0001.tgz › 692d933e-a0bf-4dcd-8f0f-7b23238f500c_Figure_S2.tif]

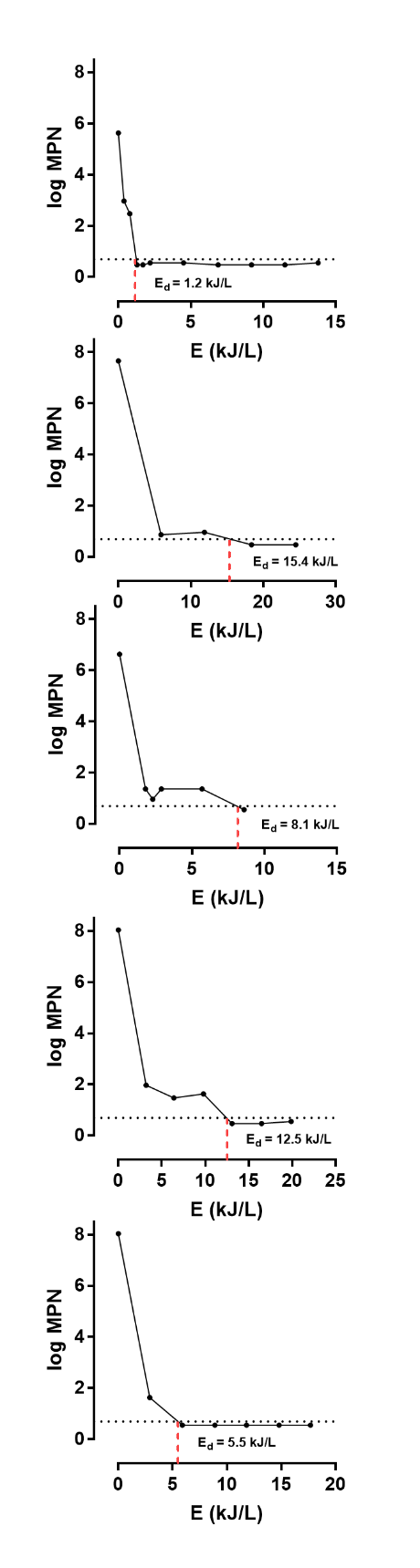

Supplement: Supplementary file 3 [file gatesopenres-2-14010-s0002.tgz › 11f610d0-8968-44d2-8e65-40b181151904_Figure_S3.tif]
