# Supplementary material for: Improving energy efficiency of electrochemical blackwater disinfection through sequential reduction of suspended solids and chemical oxygen demand
Source: Gates Open Res. 2019 Jan 23;2:50. Originally published 2018 Oct 5. [Version 2] doi: 10.12688/gatesopenres.12873.2 (PMC6350407; doi:10.12688/gatesopenres.12873.2)
Supplement: Peer review history

# Peer review: Reviewer response for version 1

**DOI:** 10.21956/gatesopenres.13962.r26807

**Published:** 2018-12-31

**Authors:** Srikanth Mutnuri

**Affiliations:** 1 Applied Environmental Biotechnology Laboratory, Department of Biological Sciences, Birla Institute of Technology and Science, Goa, India

The work focusses on minimizing the running cost of the electrochemical system by incorporating ultrafiltration and granular activated carbon. The results also show that there is reduced energy demand for running electrochemical system.

 However, it will be interesting to show the total recurring expenditure. Although there is less energy demand, it will be interesting to see the running cost for this arrangements as there will be requirement / replacement of membrane and granular activated carbon on a longer run.

 It would have been better if the authors have done repeated cycles of treatment so as to ascertain the life of the membrane and GAC.

 This seems to be a simple adsorption way of treatment! What happens to the solids that is accumulated due to membrane and GAC?

 How disinfection is proved in these experiments is not clear? Is there any special coating on the electrodes? If yes, what is it and what will be generated (hypochlorite etc) during treatment and what is the life of the coating?

 Why did the authors chose Membrane and GAC over other passive treatment techniques available?

 Can the membrane and GAC will be able to trap Helminthes eggs?

I have read this submission. I believe that I have an appropriate level of expertise to confirm that it is of an acceptable scientific standard.

# Response

**Published:** 2019-01-14

**Authors:** Brian Hawkins

**Affiliations:** Duke University, USA

**We thank Dr. Mutnuri for the review, and share his desire to learn more about the running costs and life cycles of these components and processes! Point by point comments follow.**

The work focusses on minimizing the running cost of the electrochemical system by incorporating ultrafiltration and granular activated carbon. The results also show that there is reduced energy demand for running electrochemical system.

However, it will be interesting to show the total recurring expenditure. Although there is less energy demand, it will be interesting to see the running cost for this arrangements as there will be requirement / replacement of membrane and granular activated carbon on a longer run.

**As noted in our reply to Dr. DeLong, we have greatly expanded the discussion of energy costs and the tradeoffs involved.**

It would have been better if the authors have done repeated cycles of treatment so as to ascertain the life of the membrane and GAC.

**Also as noted above, a rigorous analysis of component lifetime is underway in field tests.**

This seems to be a simple adsorption way of treatment! What happens to the solids that is accumulated due to membrane and GAC?

**In practice, GAC will need to be replaced or regenerated. Defining how frequently this needs to occur is one of the goals of the aforementioned field tests.**

**Ultrafiltration does result in a concentration of suspended solids over time in the feed tanks, which do need to be periodically cleaned out. There are several options for dealing with such a concentrated slurry, including anaerobic digestion or emptying into a containment unit that is periodically pumped out (pit, septic tank, etc.). In any case, the concentration of these solids reduces the burden of containing and transporting them significantly.**

How disinfection is proved in these experiments is not clear? Is there any special coating on the electrodes? If yes, what is it and what will be generated (hypochlorite etc) during treatment and what is the life of the coating?

**In V2 we’ve added to the Methods: “This process effects disinfection by oxidizing chloride (primarily from urine) into chlorine.”**

**And: “Bacterial inactivation was assessed with a 3-well most probable number (MPN) method using lysogeny broth (LB) for dilution and culture as previously described in detail 1-4. Disinfection was defined as reduction of MPN to < 5 / ml”**

 Why did the authors chose Membrane and GAC over other passive treatment techniques available?

**We were (and are) primarily interested in minimizing COD in the fastest and least space-intensive ways practical, since we are working towards systems for application in urban settings.**

Can the membrane and GAC will be able to trap Helminthes eggs?

**We have every reason to expect helminth eggs to be size-excluded by the membrane filtration unit, but we will be confirming this in the near future pursuant to testing to the ISO standard.**

# Peer review: Reviewer response for version 1

**DOI:** 10.21956/gatesopenres.13962.r26690

**Published:** 2018-11-06

**Authors:** Susan K. De Long

**Affiliations:** 1 Department of Civil and Environmental Engineering, Colorado State University, Fort Collins, CO, USA

This work investigated the value of ultrafiltration (UF) and GAC treatment for removal of solids and dissolved organics for application in a novel toilet based on electrochemical treatment. Although this is only a technical note, I found details lacking that made it difficult to fully appreciate the value of the work. Additionally, the authors appeared to focus only on the energy involved in the electrochemical treatment with and without the UF and GAC. A more comprehensive assessment considering the life cycle costs and energy use associated with UF and GAC would be more useful. Also, the method used to assess disinfection is very basic and may fail to detect critical classes of pathogens.

 Specific comments and suggestions: 

1. Given that disinfection is a critical aspect of this work, I suggest the authors clearly explain the methods used in the text. What media was used and what is expected to grow on this media, for example? Why not run total and fecal coliform analyses? Additionally, I am concerned that this approach does not provide a full picture of disinfection efficacy. The methods used do not provide information regarding protozoa or viruses, both of which I expect to be of critical importance. I suggest the authors specifically acknowledge these limitations in the text to avoid any miscommunication. Further, I would suggest that future studies consider a much broader range of microbial pathogens. The authors might also give consideration to whether bacteria can become viable but not culturable (VNBC) upon electrochemical treatment.
2. What is the energy and material cost associated with UF and GAC? How long can the UF and GAC filters last before they need to be replaced with normal use? How would spent GAC be handled?  
3. It would be nice to comment on the possibility of phosphorus recovery.
4. In Fig. S3, are all these data for after UF & GAC? Please clarify. It would be nice to see some comparisons with and without UF and GAC.
5. It would be helpful to more clearly explain how the electrochemical system works/will be operated such that oxidative plumbing and tanks will have less exposure to oxidative chemistry.

I have read this submission. I believe that I have an appropriate level of expertise to confirm that it is of an acceptable scientific standard.

# Response

**Published:** 2019-01-14

**Authors:** Brian Hawkins

**Affiliations:** Duke University, USA

**We greatly appreciate the thoughtful and thorough review from Dr. De Long. Point by point responses follow.**

This work investigated the value of ultrafiltration (UF) and GAC treatment for removal of solids and dissolved organics for application in a novel toilet based on electrochemical treatment. Although this is only a technical note, I found details lacking that made it difficult to fully appreciate the value of the work. Additionally, the authors appeared to focus only on the energy involved in the electrochemical treatment with and without the UF and GAC. A more comprehensive assessment considering the life cycle costs and energy use associated with UF and GAC would be more useful. Also, the method used to assess disinfection is very basic and may fail to detect critical classes of pathogens.

Specific comments and suggestions: 

1. Given that disinfection is a critical aspect of this work, I suggest the authors clearly explain the methods used in the text. What media was used and what is expected to grow on this media, for example? Why not run total and fecal coliform analyses? Additionally, I am concerned that this approach does not provide a full picture of disinfection efficacy. The methods used do not provide information regarding protozoa or viruses, both of which I expect to be of critical importance. I suggest the authors specifically acknowledge these limitations in the text to avoid any miscommunication. Further, I would suggest that future studies consider a much broader range of microbial pathogens. The authors might also give consideration to whether bacteria can become viable but not culturable (VNBC) upon electrochemical treatment.

**In V2, we have added some additional detail to the Methods. We have also added a paragraph to the Results (Now Results and Discussion) outlining our rationale for using the non-specific MPN method, and acknowledging the limitations that Dr. De Long (correctly) points out. As now noted in V2, a follow up study is planned that will include testing of specific pathogens/surrogates per the ISO 30500 standard.**

****

2. What is the energy and material cost associated with UF and GAC? How long can the UF and GAC filters last before they need to be replaced with normal use? How would spent GAC be handled?  

**In V2, we have added considerable discussion of the energy cost associated with UF. (The energy cost associated with GAC is trivial by comparison, particularly if GAC treatment is done in single pass which is our plan going forward.)**

**Assessment of component lifetime is underway. We have been field testing some GAC columns with blackwater for many months with promising results, which we hope to be publishing soon. Field tests with the specific goal of defining mean time to failure with both UF and GAC in blackwater treatment will be happening this year (2019).**

**Ultrafiltration has been applied at scale in wastewater treatment for over thirty years. An original estimate made for UF membrane life expectancy was around 7 years but the replacement frequency was lower than expected and as such longer asset lifetimes have been observed. One critical determining factor is in the extent of chemical cleaning mandated. Within this short term trial, the conversion factor, together with the trade-off between permeation rate (flux) and cross flow velocity were not optimised. These factors determine chemical cleaning frequency and the intensity of the conditions within which the membrane will be operated – both factors being a function of how hard the membrane is run (therefore limiting capital cost). Further examination of this relationship will be undertaken in future investigations to identify sustainable conditions incurring the least whole life cost, or least capital cost.**

****

3. It would be nice to comment on the possibility of phosphorus recovery.

**Evaluation of this process for phosphorus (and nitrogen) removal is underway, pursuant to testing to the ISO 30500 standard, and those data will be published in the aforementioned follow-up study.**

****

4. In Fig. S3, are all these data for after UF & GAC? Please clarify. It would be nice to see some comparisons with and without UF and GAC.

**Fig S3 is mainly meant to show how the energy of disinfection is calculated. The legend for this figure does indicate that the “…are plots of each electrochemical disinfection run following ultrafiltration and GAC treatment.”**

**We didn’t show the curves from control experiments in the supplemental figure because 1) the summary plot in Fig 1C serves the purpose of comparison, 2) there were 18 experiments in that data set, and 3) these data are already published. Further, we didn’t want to show a subset of those experiments on the XY plots in the supplemental figure because we didn’t want to give the impression that these were “matched” experiments; however, showing all of them on a single plot with more than a few curves renders the figure unintelligible.**

****

5. It would be helpful to more clearly explain how the electrochemical system works/will be operated such that oxidative plumbing and tanks will have less exposure to oxidative chemistry.

**In V2 we’ve re-written the relevant section to emphasize that we are talking about the smaller concentration-time product required to achieve disinfection: “…disinfection of pretreated blackwater is achieved with much lower free chlorine concentration-time product (CT)  (< 50 mg min / L) compared to untreated blackwater (which can require CT in excess of 2000 mg min / L to be disinfected) ^1^. This reduces the duration of time system components (plumbing, tanks) will need to be in contact with the highly oxidative chemistry of the process liquid and thus increase their service lifetime.”**
